# Supplementary material for: Cohort profile: KaroLiver, a population-based cohort of patients receiving curative liver-directed treatment for colorectal liver metastases at a Swedish tertiary centre
Source: BMJ Open. 2026 Oct 13;16(10):e125229. doi: 10.1136/bmjopen-2026-125229 (PMC13663752; doi:10.1136/bmjopen-2026-125229)
Supplement: Supplementary file 1 [file bmjopen-2026-125229.reviewer_comments.pdf]

PEER REVIEW HISTORY

BMJ Open publishes all reviews undertaken for accepted manuscripts. Reviewers are asked to complete a checklist review form and are provided with free text boxes to elaborate on their assessment. These free text comments are reproduced below.

ARTICLE DETAILS

Title (Provisional)

Cohort profile: KaroLiver, a population-based cohort of patients receiving curative liver-directed treatment for colorectal liver metastases at a Swedish tertiary centre

Authors

Gerling, Marco; Moro, Carlos Fernández; Limbecker, Celina; Viljamaa, Annika; Harrizi, Sara; Hamidi, Yousra; Hailer, Ann-Kathrin; Sterner, Johanna; Sparrelid, Ernesto; Bozóky, Lorand; Tidholm Qvist, Evelina; Baumgartner, Ruth; Schaad, Media Salmonson; Bozóky, Béla; Geyer, Natalie; Engstrand, Jennie

---

VERSION 1 - REVIEW

Reviewer **1**

Name **Huber, Tobias**

Affiliation **Department of General-, Visceral- and Transplantation Surgery, University Medical Center Mainz, Langenbeckstr. 1, 55131, Mainz, Germany**

Date **25-Aug-2026**

COI **None**

Thank you for the opportunity to review Cohort profile: KaroLiver, a population-based cohort of patients receiving curative liver-directed treatment for colorectal liver metastases at a Swedish tertiary centre by Gerling and colleagues

The cohort study reveals a large cohort of patients with CRLM that have been thoroughly analysed regarding treatment and recurrence and survival for CRLM at a single tertiary center.

I have minor comments to the manuscript which suits the cohort profile article type of the journal:

*Regarding biliary invasion: has the central bile duct been resected in theses cases? (reference to PMID: 39157729)

*It would be interesting to see how the recurrence has been treated: with resection, ablation or chemotherapy.

**Reviewer**	**2**
**Name**	**Ramouz, Ali**
**Affiliation**	**University of Heidelberg, Department of General, Visceral, and Transplantation Surgery**
**Date**	**06-Sep-2026**
**COI**	**None**

Dear editor,

With great interest, the article entitled “Cohort profile: KaroLiver, a population-based cohort of patients receiving curative liver-directed treatment for colorectal liver metastases at a Swedish tertiary centre” is reviewed. In the current study, the authors describe the establishment of a large, population-based cohort of patients who received curative-intent liver-directed treatment for colorectal liver metastases (CRLM) at a single Swedish tertiary referral centre. The cohort comprises 811 patients who underwent 1204 liver interventions between February 2012 and January 2022. The authors report a median overall survival of 51.0 months, a five-year overall survival of 44.9%, and describe a systematic programme linking clinical, radiological and whole-slide histopathological data, including annotation of tumour growth patterns at the tumour-liver interface.

Colorectal liver metastases represent a major and growing clinical burden, and the treatment landscape has evolved considerably over the past two decades, with parenchyma-sparing resection, thermal ablation and staged hepatectomy techniques expanding eligibility to increasingly complex patients. Histopathological growth patterns and molecular markers, including RAS/BRAF mutation status and mismatch repair status, are increasingly recognised as important, though still incompletely understood, determinants of recurrence and survival. Well-characterised, prospectively maintained cohorts that link detailed clinical, radiological, molecular and histopathological data are therefore of substantial value to the field, and the KaroLiver cohort is a welcome addition to a still limited number of comparable resources.

As a cohort profile, a descriptive design is appropriate and expected, and the manuscript is, on the whole, clearly written and well organised. However, several aspects of the cohort’s data completeness, variable definitions and the analytic implications of missing and clustered data would benefit from further clarification before the full value of this resource to future collaborators and secondary users can be assessed. In addition, a few passages appear to contain incomplete text or minor inconsistencies that should be corrected.

Major comments:

1. A substantial proportion of the molecular data are missing (RAS/NRAS status 53.8% missing, BRAF V600E status 56.6% missing, mismatch repair status 76.6% missing), and the manuscript attributes this to the gradual adoption of routine molecular testing over the study period. This suggests the missingness is not random, but is confounded by calendar time, so that tested and untested patients likely differ systematically in treatment era, follow-up duration

and possibly disease biology. Please discuss this more explicitly as a limitation for future molecular-stratified analyses using this cohort, rather than only reporting the proportion missing.

2. Table 3 reports histopathological variables (growth pattern, resection margin, venous/biliary invasion) at the level of 1017 resections rather than the 811 patients, since re-interventions contribute additional specimens. It is not stated how future analyses of these resection-level variables will account for patients who contribute more than one observation. Please clarify whether clustering by patient will be accounted for (eg, via mixed-effects or generalised estimating equation approaches) in downstream analyses using this variable.

3. Growth-pattern annotation, described as a core strength of the cohort, is so far complete for only 273 of 1017 resections (26.8%). Please state whether there is a plan and timeline to complete annotation of the remaining specimens, given the centrality of this variable to the cohort's stated purpose.

4. The manuscript renames the classic 'desmoplastic' growth pattern to 'encapsulated', based on the authors' own prior work, while the cited international consensus scoring guideline and most of the growth-pattern literature continue to use 'desmoplastic'. Please add an explicit cross-reference (eg, a footnote to Table 3) so that researchers comparing KaroLiver data with other published growth-pattern cohorts are not confused by this terminology change.

5. The relationship between the n=231 tumours reported in the authors' earlier growth-pattern publication (a 2012–2015 subset) and the n=273 operations / 562 tumours now annotated in KaroLiver is not explicit. Please clarify whether the earlier published subset is fully contained within the current annotated set or represents a separate batch.

6. Sex-based differences and early-onset (age <50 years) CRLM are listed as near-term research priorities for this cohort. However, Table 1 lists 'age at CRLM diagnosis' but not 'age at CRC diagnosis' as a distinct captured variable, even though early-onset status is conventionally defined by age at CRC diagnosis. This distinction will matter for the 41% of patients with metachronous disease, in whom the two ages can differ by several years. Please confirm that the underlying data model captures what is needed to classify early-onset disease correctly.

7. The term 'RAS/RAF' mutation status is used in several places (eg, the Strengths and limitations summary and the Introduction), whereas the Discussion and Tables 1–2 correctly specify 'RAS' (KRAS/NRAS) and 'BRAF' (V600E) specifically. As the RAF gene family also includes ARAF and CRAF/RAF1, which were not tested, please use 'RAS/BRAF' consistently to avoid overstating what was assessed.

8. Operating time is missing in 397 of 811 patients (49.0%), considerably higher than the proportion missing for intraoperative blood loss (21.5%), despite both typically being captured at the same operation. Please clarify the reason for this discrepancy (eg, a change in operative or anaesthesia record-keeping during the study period), given that transparent reporting of missingness mechanisms is central to the value of a cohort profile.

Minor comments:

1. In Table 2, the ‘2–3’ and ‘≥4’ metastases categories both show exactly 251 patients (30.9%). The totals reconcile with the full cohort (309+251+251=811), but please double-check that this identical value is not a data-entry or transcription artefact.

2. Table 3’s ‘Missing’ column header is formatted as ‘n = %’, while the equivalent header in Table 2 reads ‘n (%)’, matching the actual cell values shown (eg, ‘108 (10.6%)’). Please make the notation consistent between the two tables.

3. It is not explained how the N=1017 resections in Table 3 relate to the 1204 total procedures (811 first interventions + 393 re-interventions) reported in the main text; presumably the difference reflects ablation-only procedures without a resection specimen. A brief table footnote clarifying this would help readers.

4. The Author’s Contributions section ends mid-sentence (‘…JS collected and interpreted clinical data. We thank Research’), immediately followed by the AI-tools disclosure paragraph. This appears to be missing text (likely an acknowledgement of research staff) and should be completed.

VERSION 1 - AUTHOR RESPONSE

Reviewer: 1

Dr. Tobias Huber, Department of General-, Visceral- and Transplantation Surgery, University Medical Center Mainz, Langenbeckstr. 1, 55131, Mainz, Germany

Comments to the Authors:

Thank you for the opportunity to review Cohort profile: KaroLiver, a population-based cohort of patients receiving curative liver-directed treatment for colorectal liver metastases at a Swedish tertiary centre by Gerling and colleagues.

The cohort study reveals a large cohort of patients with CRLM that have been thoroughly analysed regarding treatment and recurrence and survival for CRLM at a single tertiary center.

I have minor comments to the manuscript which suits the cohort profile article type of the journal:

We thank the reviewer for constructive feedback that helped improve our manuscript.

*Regarding biliary invasion: has the central bile duct been resected in theses cases? (reference to PMID: 39157729)

Thank you for this relevant question. Biliary invasion, in this case, is a feature assessed histologically (*e.g. Reijonen et al., APMIS. 2023 Jun;131(6):249-261, Histopathological Helsinki score of colorectal liver metastases predicts survival after liver resection*), and its presence reflects invasion into the small bile ducts rather than the central bile duct. We have clarified this in the revised manuscript by adding this citation (new citation 26, Reijonen et al).

*It would be interesting to see how the recurrence has been treated: with resection, ablation or chemotherapy.

Thank you for this suggestion; we agree that the availability of these data is a strength of the cohort. In the revised manuscript, we now include an overview of the modalities used to treat recurrences with curative intent in Table 2.

Reviewer: 2
Dr. Ali Ramouz, University of Heidelberg
Comments to the Authors:
With great interest, the article entitled “Cohort profile: KaroLiver, a population-based cohort of patients receiving curative liver-directed treatment for colorectal liver metastases at a Swedish tertiary centre” is reviewed. In the current study, the authors describe the establishment of a large, population-based cohort of patients who received curative-intent liver-directed treatment for colorectal liver metastases (CRLM) at a single Swedish tertiary referral centre. The cohort comprises 811 patients who underwent 1204 liver interventions between February 2012 and January 2022. The authors report a median overall survival of 51.0 months, a five-year overall survival of 44.9%, and describe a systematic programme linking clinical, radiological and whole-slide histopathological data, including annotation of tumour growth patterns at the tumour-liver interface.

Colorectal liver metastases represent a major and growing clinical burden, and the treatment landscape has evolved considerably over the past two decades, with parenchyma-sparing resection, thermal ablation and staged hepatectomy techniques expanding eligibility to increasingly complex patients. Histopathological growth patterns and molecular markers, including RAS/BRAF mutation status and mismatch repair status, are increasingly recognised as important, though still incompletely understood, determinants of recurrence and survival. Well-characterised, prospectively maintained cohorts that link detailed clinical, radiological, molecular and histopathological data are therefore of substantial value to the field, and the KaroLiver cohort is a welcome addition to a still limited number of comparable resources. As a cohort profile, a descriptive design is appropriate and expected, and the manuscript is, on the whole, clearly written and well organised. However, several aspects of the cohort’s data completeness, variable definitions and the analytic implications of missing and clustered data would benefit from further clarification before the full value of this resource to future collaborators and secondary users can be assessed. In addition, a few passages appear to contain incomplete text or minor inconsistencies that should be corrected.

We thank the reviewer for the comprehensive and valuable review of our cohort description manuscript and have addressed the points raised as indicated below.

Majorcomments:

1. A substantial proportion of the molecular data are missing (RAS/NRAS status 53.8% missing, BRAF V600E status 56.6% missing, mismatch repair status 76.6% missing), and the

manuscript attributes this to the gradual adoption of routine molecular testing over the study period. This suggests the missingness is not random, but is confounded by calendar time, so that tested and untested patients likely differ systematically in treatment era, follow-up duration and possibly disease biology. Please discuss this more explicitly as a limitation for future molecular-stratified analyses using this cohort, rather than only reporting the proportion missing.

The reviewer rightly points out that mutational testing is non-random in clinical practice and changes systematically over time. We included this as a limitation in the Strengths and Limitations section of the original manuscript, and to address the reviewer's point, we have expanded it further as suggested in the same section.

2. Table 3 reports histopathological variables (growth pattern, resection margin, venous/biliary invasion) at the level of 1017 resections rather than the 811 patients, since re-interventions contribute additional specimens. It is not stated how future analyses of these resection-level variables will account for patients who contribute more than one observation. Please clarify whether clustering by patient will be accounted for (eg, via mixed-effects or generalised estimating equation approaches) in downstream analyses using this variable.

   Thank you for this observation, which raises an interesting analytical point. Handling resection-level variables will depend on the specific research question. For example, when relating tumour-intrinsic features to one another, such as growth patterns and venous invasion, a resection-level analysis may be appropriate. In contrast, when relating stable patient-level characteristics, such as sex, to variable features, repeated observations within patients will need to be accounted for, as suggested by the reviewer. We have added a statement clarifying this in the "Future Plans" section.

3. Growth-pattern annotation, described as a core strength of the cohort, is so far complete for only 273 of 1017 resections (26.8%). Please state whether there is a plan and timeline to complete annotation of the remaining specimens, given the centrality of this variable to the cohort's stated purpose.

We have added a sentence to clarify this in the Future Plans section. Our aim is to complete growth pattern scoring for all patients as soon as possible.

4. The manuscript renames the classic 'desmoplastic' growth pattern to 'encapsulated', based on the authors' own prior work, while the cited international consensus scoring guideline and most of the growth-pattern literature continue to use 'desmoplastic'. Please add an explicit cross-reference (eg, a footnote to Table 3) so that researchers comparing KaroLiver data with other published growth-pattern cohorts are not confused by this terminology change.

Thank you for pointing this out. To clarify the terminology change, we added a statement and the reference describing the suggested change in detail to the "Histology" section.

5. The relationship between the n=231 tumours reported in the authors' earlier growth-pattern

publication (a 2012–2015 subset) and the n=273 operations / 562 tumours now annotated in KaroLiver is not explicit. Please clarify whether the earlier published subset is fully contained within the current annotated set or represents a separate batch.

Thank you for pointing this out. In response, we added a reference to the 2023 paper, which, as the reviewer notes, describes a subset of patients included in KaroLiver, in the "Findings to date" section to clarify this. Tumors with GP scores in the 2023 publication do comprise a subset, as indicated in the original manuscript, and we have now identified the related publication more clearly.

6. Sex-based differences and early-onset (age <50 years) CRLM are listed as near-term research priorities for this cohort. However, Table 1 lists 'age at CRLM diagnosis' but not 'age at CRC diagnosis' as a distinct captured variable, even though early-onset status is conventionally defined by age at CRC diagnosis. This distinction will matter for the 41% of patients with metachronous disease, in whom the two ages can differ by several years. Please confirm that the underlying data model captures what is needed to classify early-onset disease correctly.

Thank you for this suggestion. We added "date of CRC resection" to Table 1, as it is routinely collected in KaroLiver. This variable is included for all eligible patients and allows the calculation of the patient's age at CRC diagnosis and at CRC resection. Consequently, the dataset can reliably support research questions on early-onset CRC based on age at CRC diagnosis, as suggested by the reviewer.

7. The term 'RAS/RAF' mutation status is used in several places (eg, the Strengths and limitations summary and the Introduction), whereas the Discussion and Tables 1–2 correctly specify 'RAS' (KRAS/NRAS) and 'BRAF' (V600E) specifically. As the RAF gene family also includes ARAF and CRAF/RAF1, which were not tested, please use 'RAS/BRAF' consistently to avoid overstating what was assessed.

Where applicable, we changed the term "*RAS/RAF*" to "*RAS/BRAF*" in the text and figures. We agree that "*BRAF*" is the more clinically relevant term, although *ARAF/RAF1* are analyzed in more recent panels, including the GMCK panel used at our hospital (Salgkamis et al., Sci Rep. 2024 Aug 6;14(1):18275. doi: 10.1038/s41598-024-69285-8).

8. Operating time is missing in 397 of 811 patients (49.0%), considerably higher than the proportion missing for intraoperative blood loss (21.5%), despite both typically being captured at the same operation. Please clarify the reason for this discrepancy (eg, a change in operative or anaesthesia record-keeping during the study period), given that transparent reporting of missingness mechanisms is central to the value of a cohort profile.

Thank you for this relevant observation. This discrepancy in missingness reflects differences in documentation practices. In our electronic health records system, intraoperative blood loss is regularly documented directly in the operation report, whereas operating time is documented less frequently, resulting in a higher proportion

of missing operating time data than blood loss.

Minor comments:

1. In Table 2, the ‘2–3’ and ‘≥4’ metastases categories both show exactly 251 patients (30.9%). The totals reconcile with the full cohort (309+251+251=811), but please double-check that this identical value is not a data-entry or transcription artefact.

We thank the reviewer for this observant comment. We revisited the dataset and verified these values. We confirm that both categories contain 251 patients (30.9%) each, and that these identical counts are correct rather than the result of a data-entry or transcription error.

2. Table 3’s ‘Missing’ column header is formatted as ‘n = %’, while the equivalent header in Table 2 reads ‘n (%)’, matching the actual cell values shown (eg, ‘108 (10.6%)’). Please make the notation consistent between the two tables.

Thank you for bringing this to our attention. We changed the heading of Table 3 from “n = %” to “n (%)”, making it consistent with the heading of Table 2 and the format of the cell values of the respective column. Please note that the former Table 3 became Table 4 in the revised manuscript.

3. It is not explained how the N=1017 resections in Table 3 relate to the 1204 total procedures (811 first interventions + 393 re-interventions) reported in the main text; presumably the difference reflects ablation-only procedures without a resection specimen. A brief table footnote clarifying this would help readers.

We agree and, as suggested, we have added a footnote to clarify this.

4. The Author’s Contributions section ends mid-sentence (‘…JS collected and interpreted clinical data. We thank Research’), immediately followed by the AI-tools disclosure paragraph. This appears to be missing text (likely an acknowledgement of research staff) and should be completed.

Thank you for catching this oversight, which resulted from splitting author contributions and acknowledgments from a previous manuscript version. We have removed the half-sentence.

VERSION 2 - REVIEW

Reviewer **2**

Name **Ramouz, Ali**

Affiliation **University of Heidelberg, Department of General, Visceral, and Transplantation Surgery**

**Date**	**29-Sep-2026**
**COI**	**None**

Dear editor,

I thank the authors for their careful and thorough revision. They have addressed all of my previous comments. In particular, the expanded limitation on molecular data, the clarification on the "encapsulated" terminology, the addition of date of CRC diagnosis to Table 1, and the consistent use of "RAS/BRAF" all improve the manuscript. I have only a few minor points left.

1. Missingness of operating time (previous major comment 8). The response letter explains why operating time is missing more often than blood loss (documentation practice in the electronic health record). This explanation is not in the manuscript. Please add one sentence, for example to the Results or as a footnote to Table 3, because the reasons data are missing matter to future users of the cohort.

2. The earlier growth-pattern subset (previous major comment 5). The Findings to date section now describes the earlier subset as "n = 231 patients", but the previous version said "n = 231 tumours". Please check which unit is correct against reference 5. Please also state whether this subset is fully contained in the 273 annotated operations. Relatedly, "within the next years" is not a firm timeline for completing growth-pattern annotation. An approximate target would help researchers planning collaborative work.

3. Molecular testing bias. The authors now note that molecular testing in the early years was done mostly in patients who later received palliative treatment. This means testing was linked to outcome, not just to calendar period. In early-era analyses, patients with molecular data are therefore likely to be enriched for recurrence and poorer prognosis. A brief sentence making this explicit would strengthen the limitation.

4. Table 4. The growth-pattern row gives percentages "of patients with available growth pattern", but the denominator (n = 273) counts operations, not patients. Please correct the label. The wording of footnote 2 could also be clearer, and stating the number of ablation-only procedures excluded (1204 − 1017 = 187) would reconcile the totals explicitly.

VERSION 2 - AUTHOR RESPONSE

Reviewer: 2

Comments to the Authors:

I thank the authors for their careful and thorough revision. They have addressed all of my previous comments. In particular, the expanded limitation on molecular data, the clarification on the "encapsulated" terminology, the addition of date of CRC diagnosis to Table 1, and the consistent use of "RAS/BRAF" all improve the manuscript. I have only a few minor points left.

1. Missingness of operating time (previous major comment 8). The response letter explains why operating time is missing more often than blood loss (documentation practice in the electronic health record). This explanation is not in the manuscript. Please add one sentence, for example to the Results or as a footnote to Table 3, because the reasons data are missing matter to future users of the cohort.

In response, we added this information as a footnote to Table 3.

2. The earlier growth-pattern subset (previous major comment 5). The Findings to date section now describes the earlier subset as "n = 231 patients", but the previous version said "n = 231 tumours". Please check which unit is correct against reference 5. Please also state whether this subset is fully contained in the 273 annotated operations. Relatedly, "within the next years" is not a firm timeline for completing growth-pattern annotation. An approximate target would help researchers planning collaborative work.

We thank the reviewer for pointing this out. Indeed, we noticed this oversight during revision and corrected the statement from "tumors" to "patients," as described previously in Fernández Moro et al., 2023. This subset is fully contained in the currently included 273 resections.

Future growth pattern scoring depends on several steps we cannot fully control, such as pathology retrieval, scanning, and case complexity, which is why we cannot give a definitive timeline in the manuscript. Nevertheless, we have begun this process and have therefore amended the text to state a timely of one to two years.

3. Molecular testing bias. The authors now note that molecular testing in the early years was done mostly in patients who later received palliative treatment. This means testing was linked to outcome, not just to calendar period. In early-era analyses, patients with molecular data are therefore likely to be enriched for recurrence and poorer prognosis. A brief sentence making this explicit would strengthen the limitation.

We added an additional sentence addressing this issue in the "Strengths and limitations" section.

4. Table 4. The growth-pattern row gives percentages "of patients with available growth pattern", but the denominator (n = 273) counts operations, not patients. Please correct the label. The wording of footnote 2 could also be clearer, and stating the number of ablation-only procedures excluded (1204 − 1017 = 187) would reconcile the totals explicitly.

We are thankful for this in-depth comment. The phrasing "of patients with available growth pattern" in the Table column is misleading; it should read, "of resections with available growth pattern." These resections also included non-index resections, and excluded ablations, which explains the differences in the number of patients between the tables, and is made in order to present all available data for the histological variables specifically. We updated our manuscript accordingly and modified the footnote to state this difference more clearly for the interested reader. We feel that adding ablation

numbers to this table would be rather confusing for most readers, and would feel the current description to be both accurate and sufficient for most contexts.
